# Two New Withanolide Lactones from Flos Daturae

**DOI:** 10.3390/molecules16075833

**Published:** 2011-07-11

**Authors:** Hai-Xue Kuang, Bing-You Yang, Yong-Gang Xia, Qiu-Hong Wang

**Affiliations:** Key Laboratory of Chinese Materia Medica, Heilongjiang University of Chinese Medicine, Ministry of Education, Harbin 150040, China

**Keywords:** Flos Daturae, *Datura metel* L., withanolide lactones

## Abstract

Chemical investigation of the 50% ethanol eluate fraction from a macroporous resin of flowers of *Datura metel* L. collected in the Jiangsu Province of China resulted in the isolation of two new withanolides, baimantuoluoline G (**1**) and baimantuoluoside H (**2**). Their structures were elucidated as (12*β*,6*β*,22*R*)-1,10-seco-6,12,27-trihydroxy-26-oxo-witha-3,5,24-trienolide-1-oic acid-*ε*-lactone (**1**) and (5*β*,6*α*,12*β*,22*R*)-5,6,12,27-tetra-hydroxy-1,26-dioxo-witha-2,24-dienolide-27-O-*β-*glucopyranoside (**2**) on the basis of extensive spectroscopic analysis (1D, 2D-NMR and HRESIMS) and chemical studies.

## 1. Introduction

**Figure 1:**
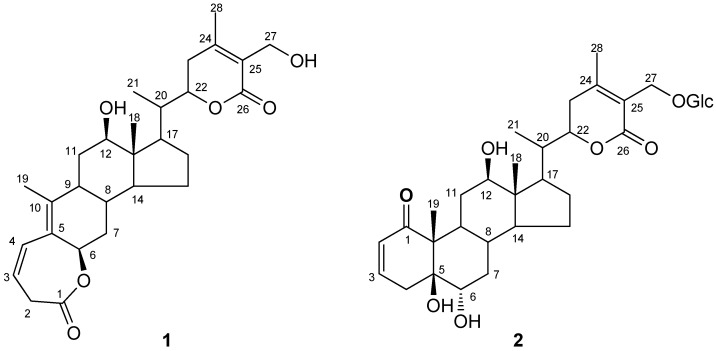
Structures of **1** and **2**.

Flos Daturae is the dry flower of *Datura metel* L. (Solanaceae), which widely distributed in China [1]. Flos Daturae, known as “baimantuoluo” or “yangjinhua” in China, has been used for centuries in Traditional Chinese Medicine for the treatment of asthma, convulsions, pain, and rheumatism [2]. Flos Daturae has been found to be rich in tropane alkaloids [2]. Besides, a number of withanolides have also been isolated from Flos Daturae [3,4,5,6,7,8]. Recently Flos Daturae has been used clinically for the treatment of psoriasis in China [9]. The effective part for psoriasis, namely the non-alkaloid water-soluble fraction of Flos Daturae, has been demonstrated to have anti-inflammatory, anti- skin titillation and anti-anaphylaxis actions by detailed pharmacological experiments [9]. However, its active constituents and pharmacological effects related to the treatment of psoriasis were not fully elucidated. As a part of a continuing project to study the active constituents of Flos Daturae for psoriasis [6,7,8], we investigated the 50% ethanol eluate fraction from a macroporous resin of the flowers of *D. metel*. Our extraction and separation method can greatly enrich fractions in withanolide compounds so trace withanolides can be isolated. In this paper, we present the isolation and structural characterization of the two new withanolide lactones (Figure 1) on the basis of the interpretation of spectral data, including 1D, 2D NMR and HRESIMS data. A 1,10-seco withanolide is reported from only the third time in herb plants.

## 2. Results and Discussion

Compound **1** was obtained as a white amorphous powder. Its molecular formula was established as C_28_H_38_O_6_ by the positive HRESIMS, indicating six degrees of unsaturation. The ^1^H-NMR spectrum of **1** (Table 1), showed several characteristic signals of the common withanolide steroid. Three signals at *δ* 0.78 (3H, *s*), 1.18 (3H, *d*, *J* = 6.8 Hz), 1.84(3H, *s*) and 2.10 (3H, *s*) were attributed to Me-18, Me-21, Me-19 and Me-28, respectively. A Me-27 signal was missing, and was replaced by two doublets at *δ* 4.37 (1H, *d*, *J* = 11.7 Hz) and 4.30 (1H, *d*, *J* = 11.7 Hz), suggesting that C-27 was substituted by hydroxyl group. The double doublet at *δ* 3.47 (1H, *dd*, *J* = 10.9, 4.5 Hz) was characteristic for a 12*β*-hydroxywithanolide [4]. H-22 resonated as a double triplet at *δ* 4.62 (1H, *dt*, *J* = 12.9, 3.4 Hz), revealing a *R* configuration at C-22 [5,6].

**Figure 2:**
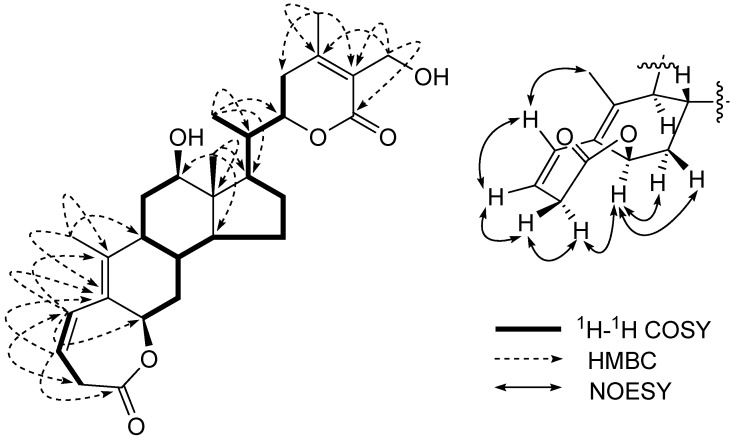
Key ^1^H-^1^H COSY and HMBC correlations of **1**.

The ^13^C-NMR spectrum of **1** showed resonances for all 28 carbons. The characteristic downfield signal at *δ* 175.5 was due to two lactone carbonyls, respectively, along with the characteristic doublets at *δ* 126.3, 157.9 and 168.6 were attributed to C-24, C-25 and C-26 of the *α,β*-unsaturation-*γ*-lactone ring respectively, in the ring E. The signals at *δ* 118.1, 130.2, 126.5 and 142.5 for the vinylic carbons at C-3, C-4, C-5, C-10 respectively, in the ring A. The typical signals at *δ* 74.8, 78.6, 80.8, and 56.3 were assigned to the oxygenated carbons at C-6, C-12, C-22 and C-27, respectively. The signals appearing at *δ* 8.1, 15.6, 15.3 and 20.2 were assigned to the Me-18, Me-19, Me-21 and Me-28, respectively. In addition, a signal at *δ*_H_ 5.30 (1H, *br. s*) together with the signal in the ^13^C NMR spectrum of a keto-carbonyl of C-1 upfield of *δ*_C_ 175.5 due to an ester group of CO-O-CH indicated that **1 **possesses a 1,10-*seco*-steroid skeleton [10,11]. Further support this assumption was obtained from the fact that the methylene protons at C-2 [*δ* 4.03 (1H, *br. d*, *J* =17.4 Hz) and 3.08 (1H, *dd*, *J* =17.4, 8.7 Hz)] were unusually shifted to a low field, suggesting the methylene to be situated between an ester carbonyl group and carbon-carbon double bond. Based on this finding and HMBC correlations, between C-1 (*δ*_C_ 175.5) and H-2*α*, H-2*β*, and H-3, between H-4 (6.62 (1H, *dd*, *J = *11.4, 3.1 Hz) and C-2, C-5, C-6, and C-10, and between C-5 and Me-19, as shown in Figure 2. Thus, a seven-membered *β*, *γ*-unsaturated lactonic moiety was determined in ring A. 

The *β*-configuration of the lactone bond at C-6 was established by a NOESY experiment (Figure 2). The NOESY spectrum showed the correlation between H-6 (1H, *br. s*) and H-2*α*, H-7*α *and H-7*β*, indicating that H-6 has the same configuration as H-2*α*. Since a small coupling between H-2*α *and H-3 in the ^1^H NMR spectrum was observed due to an approximate 90°, a *β*-configuration of the lactone bond at C-6 was inferred, which is in agreement with those of 1,10-seco steroids [10,11]. Therefore, the structure of **1** was deduced as (12*β*,6*β*,22*R*)-1,10-seco-6,12,27-trihydroxy-26-oxo-witha-3,5,24-trienolide-1-oic acid-*ε*-lactone, which was named baimantuoluoline G.

**Table 1:** ^1^H and ^13^C-NMR data of **1** and **2** in CD_3_OD at 400 MHz and 100 MHz, *J *in Hz.

No.	1		2	
	*δ*_H_	*δ*_C_	*δ*_H_	*δ*_C_
1		175.5		207.0
2	4.03 (1H, *br. d*, *J* = 17.4, *α*-H)	35.9	5.77 (1H, *dd*, *J* = 10.0, 2.4)	128.9
	3.08 (1H, *dd*, *J* = 17.4, 8.7, *β*-H)			
3	5.53 (1H, *dd*, *J* = 11.4, 8.7)	118.1	6.66 (1H, *ddd*, *J* = 10.0, 5.2, 2.0)	143.5
4	6.62 (1H, *dd*, *J* = 11.4, 3.1)	130.2	3.24 (1H, *dt*, *J* = 20.0, 2.4)	36.5
			2.05 (1H, *dd*, *J* = 20.0,5.2)	
5		126.5		78.2
6	5.30 (1H, *br. s*)	74.8	3.52 (1H, *t*, *J* = 2.0)	75.2
7	2.03 (1H, *m*)	33.7		33.8
	1.43 (1H, *m*)			
8	1.50 (1H, *m*)	33.5		30.5
9	1.77 (1H, *m*)	47.2	1.87 (1H, *m*)	41.0
10		142.5		52.8
11	2.14 (1H, *dt*, *J* = 16.1, 4.2)	36.9	2.42 (1H, *dt*, *J* = 12.4, 4.0)	33.8
	1.36 (1H, *m*)		1.36 (1H, *m*)	
12	3.47 (1H, *dd*, *J* = 10.9, 4.5)	78.6	3.47 (1H, *dd*, *J* = 11.2, 4.4)	78.7
13		49.1		49.0
14	1.25 (1H, *m*)	53.8	1.25 (1H, *m*)	55.3
15	1.72 (1H, *m*)	23.8	1.76 (1H, *m*)	24.6
	1.30 (1H, *m*)		1.31 (1H, *m*)	
16	1.73 (1H, *m*)	27.7	1.76 (1H, *m*)	27.6
	1.60 (1H, *m*)		1.54 (1H, *m*)	
17	1.61 (1H, *m*)	54.5	1.56 (1H, *m*)	55.0
18	0.78 (3H, *s*)	8.1	0.76(3H, *s*)	8.0
19	1.84 (3H, *s*)	15.6	1.30(3H, *s*)	16.1
20	2.01 (1H, *m*)	38.9	2.05 (1H, *m*)	38.9
21	1.18 (3H, *d*, *J* = 6.8)	15.3	1.18 (3H, *d*, *J* = 6.8)	15.5
22	4.62 (1H, *dt*, *J* = 12.9, 3.4)	80.8	4.60 (1H, *dt*, *J* = 13.6, 4.0)	80.9
23	2.55 (1H, *dd*, *J* = 18.0, 13.2)	32.5	2.58 (1H, *dd*, *J* = 18.0, 13.2)	32.3
	2.24 (1H, *dd*, *J* = 18.0, 3.2)		2.21 (1H, *dd*, *J* = 18.0, 3.2)	
24		157.9		160.5
25		126.3		123.6
26		168.6		168.7
27	4.37 (1H, *d*, *J* = 11.7)	56.3	4.46 (1H, *d*, *J* = 10.8)	63.5
	4.30 (1H, *d*, *J* = 11.7)		4.61 (1H, *d*, *J* = 10.8)	
28	2.10 (3H, *s*)	20.2	2.13 (3H, *s*)	20.8
1′			4.31 (1H, *d*, *J* = 8.0)	103.9
2′			3.16 (1H, *t*, *J* = 8.0)	75.0
3′			3.26 (1H, *m*)	78.0
4′			3.29 (1H, *m*)	71.5
5′			3.24 (1H, *m*)	78.0
6′			3.85 (1H, *dd*, *J* = 12.0, 2.0)	62.7
			3.67 (1H, *dd*, *J* = 12.0, 5.2)	

Compound **2** was obtained as a white amorphous powder and showed positive results for the *Molish* reagent, which was indicative of a withanolide glycoside. Its molecular formula was established as C_34_H_50_O_1__2_ by the positive HRESIMS, indicating 10 degrees of unsaturation. The ^1^H-NMR spectrum of **2** showed distinct resemblance to those of baimantuoluoside G ((5*α*,6*β*,22R)-5,6,27-trihydroxy-1-oxowitha-2,24-dienolide-27-O*-β-*glucopyranoside) [6]. The only notable difference was the change of H-12 signal appeared as a double doublet at *δ* 3.47 (1H, *dd*, *J* = 11.2, 4.4 Hz), indicating that C-12 was substituted by a hydroxyl group. The ^13^C-NMR (DEPT) spectrum showed an additional downfield C-atom signal at *δ* C 78.7 in **2**, which was affirmatively assigned to the C-12. On the basis of above data, the structure of **2 **was identified to be (5*β*,6*α*,12*β*,22*R*)-5,6,12,27-tetrahydroxy-1,26-dioxo-witha-2,24-dienolide-27-O-*β-*glucopyranoside, and was named baimantuoluoside H.

## 3. Experimental

### 3.1. General

Preparative HPLC (Waters, Delta 600-2487) was performed on a Hypersil-ODS II (10 μm, 20 × 300 mm, Yilite, Dalian, People’s Republic of China) with Waters Empower software. IR spectra were measured in KBr discs on a Shimadzu FTIR-8400S spectrometer. HRESIMS were carried out on Waters Xevo QTOF mass spectrometer with Masslynx V4.11 software. NMR data were recorded in CD_3_OD on Bruker DPX 400 spectrometer at 400 MHz for (^1^H) and 100 MHz (^13^C) with Xwin-NMR software version 2.6; chemical shifts *δ* in ppm rel. to SiMe_4_ as internal standard, coupling constant *J* in Hz. The pulse conditions were as follows: for the ^1^H-NMR spectra, spectrometer frequency (SF) 400.13 MHz, acquisition time (AQ) 2.0447731s, number of transients (NS) 64, receiver gain (RG) 128, temperature (TE) 303.0 K, dwell time (DW) 62.400 μs, per scan delay (DE) 7.00 μs, dummy scans (DS) 0; for the ^13^C-NMR spectrum, SF 100.62 MHz, AQ 0.65 s, NS 1430, RG 14596.5, TE 303.0 K, DW 19.900 μs, DE 28.00 μs, DS 0; for the COSY spectrum, SF 400.13 MHz, NS 16, DS 16, pulse (P1) 10.8 μs, TE 303.0 K, RG 574.7, DW 170.400 μs, DE 7.00 μs; for the NOE experiments, SF 400.13 MHz, NS 64, DE 7.00 μs; for the HMBC spectrum, SF 400.13 MHz, AQ 0.1745396 s, RG 16384, NS 64, DW 170.400 μs, DS 16, DE 7.00 μs, TE 303.0 K; and for the HSQC spectrum, SF 400.13 MHz, AQ 0.1745396 s, NS 32, DS 16, DE 7.00 μs, DW 170.400 μs, RG 16384, TE 303.0 K.

### 3.2. Plant Material

The dry flowers of *D. metel *were collected in Nanjing city of Jiangsu Province of China in September 2002, and identified by Prof. Zhenyue Wang. A voucher specimen (No. 2002035) is deposited at the Herbarium of Heilongjiang University of Chinese Medicine, China.

### 3.3. Extraction and Isolation

The dried flowers (30 kg) of *D. metel *L*. *were extracted with 70% EtOH under reflux (2 × 100 L) for 2.5 h (each), and the combined solution was filtered and evaporated under vacuum to a syrup (45 °C), followed by suspension in H_2_O (500 L). The suspension was acidified with 0.1% HCl, and then filtered and exchange for Styrene-DVB (001×7). The exchanged solution was passed through AB-8 crosslinked polystyrene, and sequentially eluted with H_2_O, 50% EtOH, and 95% EtOH, respectively. 50% EtOH elution was concentrated under vacuum to yield a syrup (52.0 g) and this crude residue was subjected to silica gel (300 mesh, 50 × 8 cm, flow rate 10 mL/min) and eluted successively with 6 L of CHCl_3_/MeOH during each gradient (10:1→1:1) to give 10 fractions (Fr. 1–10). Fr. 7 (5 g) continues silica gel chromatography (300 mesh, 10 × 2 cm, flow rate 1 mL/min) eluted with 600 mL of CHCl_3_/MeOH during each gradient (5:1→1:1) to afford a number of sub-fractions A_1_-A_13_. Compounds **1** (15 mg, *t*_R_ = 37.2 min) and **2 **(21 mg, *t*_R_ = 12.3 min) were obtained from the sub-fraction A_4_ (0.9 g) with MeOH/H_2_O (2:3) by prep. HPLC chromatography on a Hypersil-ODS II column (10 μm, 20 × 300 mm, flow rate 8 mL/min).

*Baimantuoluoline G* (**1**). White amorphous powder, [α]^25^_D_ = +17.0 (c = 0.1, MeOH). IR (KBr): ν_max_ = 3426, 2925, 1708, 1387, 1286, 1135, 1089, 995 cm^−1^. HRESIMS (positive): *m/z *= 493.2578 (calc. for C_28_H_38_NaO_6_, 493.2566, [M+Na]^+^) and 509.2327 (calc. for C_28_H_38_KO_6_, 509.2305, [M+K]^+^). ^1^H- and ^13^C-NMR: see Table 1.

Baimantuoluoside H (2): White amorphous powder, [α]^25^_D_ = +24 (c = 0.1, MeOH). IR (KBr): ν_max_ = 3411, 3326, 2942, 2923, 2865, 2590, 1670, 1070, 1030 cm^−1^. HRESIMS (positive): *m/z = *673.3224 (calc. for C_34_H_50_NaO_12_, 673.3200, [M+Na]^+^) and 689.2948 (calc. for C_34_H_50_KO_12_, 689.2939, [M+K]^+^). ^1^H- and ^13^C-NMR: see Table 1.

## 4. Conclusions

Withanolide lactones constitute a group of C28 steroidal lactones isolated from several genera of Solananceae [3]. A characteristic feature of their skeleton is the (mostly) *α*,*β*-unsaturated *δ*-lactone ring formed in the side chain [3], which has been reported to be associated with diverse biological activities including cytotoxic, anti-inflammatory, antioxidant, and antitumor properties [4]. As a part of our chemical investigation on *D. metel*, we have isolated two new withanolide lactones. Their structures were established on the basis of spectroscopic evidence. Compound **1 **is a novel withanolide lactone obtained from a *D. metel *with an unusual seven-membered *β*, *γ*-unsaturated lactone of ring A.
